# Identification of a *MIP* mutation that activates a cryptic acceptor splice site in the 3′ untranslated region

**Published:** 2010-11-02

**Authors:** Chongfei Jin, Jin Jiang, Wei Wang, Ke Yao

**Affiliations:** 1 Eye Center of the Second Affiliated Hospital, Medical College of Zhejiang University, Hangzhou, China; 2 Department of Ophthalmology, Zhejiang Provincial People’s Hospital, Hangzhou, China

## Abstract

**Purpose:**

To investigate the consequence of a major intrinsic protein *MIP* splice-site mutation (c.607–1G>A) in a four-generation Chinese pedigree afflicted with autosomal dominant congenital cataracts (ADCC).

**Methods:**

Both a mutated minigene with c.607–1G>A, and a wild-type minigene were constructed using the pTARGET^TM^ mammalian expression vector. They were transiently transfected into HeLa cells and human lens epithelial cells, respectively. After 48 h incubation, RNA extraction and RT–PCR analysis were performed and PCR products were separated and confirmed by sequencing. Structural models of the wild-type and the mutant aquaporin 0 (AQP0) were generated and analyzed using SWISS-MODEL.

**Results:**

The G>A transition activated a cryptic acceptor splice site (c.965–966) in the 3′ untranslated region (3′ UTR), resulting in the absence of the coding region and most of the 3′UTR in exon 4 of the mature mRNA. Moreover, homology modeling of the mutant protein suggested that the sixth transmembrane helix and carboxyl terminus were replaced with the Leu-His-Ser tripeptide (AQP0-LHS).

**Conclusions:**

The *MIP* splice-site mutation (c.607–1G>A) activates a cryptic acceptor splice site in the 3′ UTR, which may result in substitution of the sixth transmembrane helix and carboxyl terminus for AQP0-LHS. To our knowledge, this is the first report of activation of a cryptic splice site in the 3′ UTR in a human disease gene.

## Introduction

Hereditary congenital cataract (OMIM 604307) is an opacification of the eye lens which presents at birth or shortly thereafter. Despite the great advances in understanding of lens structure and function, the relationships among cataract morphology, etiology, and pathologic mechanisms are still unclear. Congenital cataracts remain the leading cause of visual disability in children worldwide [1-4]. In 2000, the major intrinsic protein (MIP, MP26, AQP0) was reported to be associated with autosomal dominant congenital cataract (ADCC) [5]. To date, seven mutations in the *MIP* gene have been identified from seven unrelated human families; five missense mutations (T138R, E134G, R33C, R233K, and V107I) [5-8], one deletion mutation causing a frameshift that alters 41 of 45 subsequent amino acids and creates a premature stop codon (G213VfsX45) [9], and one splice-site mutation causing autosomal dominant congenital cataract (ADCC) with “snail-like” phenotype in a large Chinese family (our laboratory’s recently reported finding, c.607–1G>A) [10].

The effect of mutations that alter critical amino acids and generate premature stop codons can be predicted from triplet codon analysis. However, it is difficult to predict the effects of changes in intronic sequences. The production of mature mRNA requires an accurate and efficient removal of introns from pre-mRNA by the spliceosome [11]. The regulation of alternative splicing usually relies on a choice from among candidate splice sites, either alternative 5′ or alternative 3′ sites. Mutations can disrupt splicing by directly inactivating or creating an alternative splicing site, by activating a cryptic splice site or by interfering with splicing regulatory elements. Although the activation of cryptic splice sites has been reported as a mechanism in human disease, to the best of our knowledge currently there is no report of activation of cryptic splice site in the 3′ untranslated region (3′ UTR, sequences on the 3′ end of mRNA which are not translated into protein) [12].

## Methods

### Plasmid and minigene constructs

**Figure 1:**
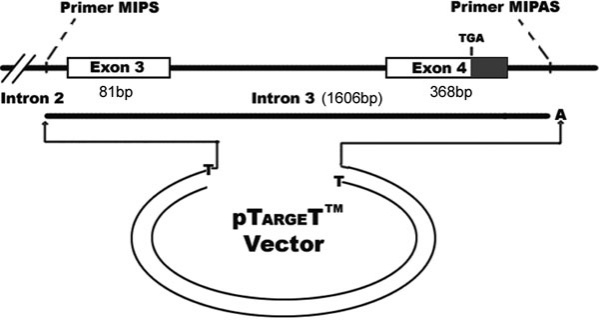
Schematic diagram of the minigene construct. It was obtained by insertion of a PCR fragment containing the genomic *MIP* sequence into the pTARGET mammalian expression vector after the A-tail was added. It consists of the 5′ flanking sequence of exon 3, exon 3, intron 3, and exon 4 including the coding and the 3-untranslated region (3′UTR) which was amplified using primers MIPS and MIPAS.

A mutated minigene was obtained by the insertion of a PCR fragment containing the genomic *MIP* sequence from the proband into the pTARGET^TM^ mammalian expression vector (Promega, Milan, Italy). It consisted of the 5′ flanking sequence of exon 3, exon 3, intron 3, and exon 4 including the coding and the 3-untranslated region (3′UTR) which was amplified using primers MIPS (5′-TTG ACC CCA AGG TAG AAA TGA CG-3′) and MIPAS (5′-ACA ATC AGC ACA CAC GGC GAA G-3′). PCR was performed on 100 μg genomic DNA in a standard 50 μl volume, containing 1.25 PrimeSTAR^®^ HS DNA Polymerase (Takara Bio, Otsu, Japan). The cycling conditions for PCR were 35 cycles of 98 °C for 10 s, 68 °C for 4 min, preceded by 30 s at 98 °C and followed by a final elongation step at 68 °C for 10 min. The PCR product was cloned into the vector after the addition of an A-tail (Figure 1). The addition of the A-tail was achieved by incubating 5 μl purified PCR product in a 10 μl volume, containing dATP (final concentration 0.2 mM) and 5 U Taq DNA Polymerase (Takara Bio), The temperature condition was at 70 °C for 30 min. Plasmid DNA was isolated using a Miniprep Kit (Axygene, Union City, CA) and *MIP* c.607–1G>A was ascertained by sequencing—pTARGET-mut. A similar approach was used to obtain the corresponding wild type minigene by amplifying the genomic DNA from a healthy individual—pTARGET-wt. Each clone was entirely sequenced to confirm that no other mutations had been introduced by the PCR procedure.

### Cell culture and transient transfection

HeLa cells and human lens epithelial cells (LECs, HLE B-3) were cultured in RPMI 1640 supplemented with 10% fetal bovine serum. Cells were grown in a humidified atmosphere of 5% CO_2_ and 95% air at 37 °C and cultured according to standard procedures. Transient transfections were performed in 10-cm dishes (2×10^6^cells/dish) with Lipofectamine™ 2000 (Invitrogen, Carlsbad, CA) using 4 µg of total plasmid DNA Endofree purified (Qiagen, Hilden, Germany) following the manufacturer’s instructions.

### Minigene splicing assay

Both HeLa cell and LECs were transfected with pTARGET-mut and pTARGET-wt minigene constructs. The blank minigene construct pTARGET-con was used as a control. Forty-eight hours after transfection, total RNA from the cells was extracted with an RNeasy Mini Kit (Qiagen) and analyzed by RT–PCR. Reverse transcription was performed using M-MuLV reverse transcriptase (Fermentas, Vilnius, Lithuania) and oligo (dT) primer in a 20 μl volume according to the protocol of the manufacturer, and the PCR reaction was performed with a forward primer (5′-CTT TGC TCC TGC CAT TCT-3′) and a vector-specific reverse primer (5′-GGC TTT ACA CTT TAT GCT TC-3′). PCR products were separated by electrophoresis on a 2% agarose gels and confirmed by sequencing.

### Comparative modeling of AQP0

The three-dimensional structure of AQP0 was modeled on the basis of the crystal structure of bovine AQP0. The homology model was generated by SWISS-MODEL and analyzed in the Swiss-PdbViewer, Version 3.7 [13-15].

## Results

### Effect of the intronic mutation on the processing of the pre-mRNA transcript

**Figure 2:**
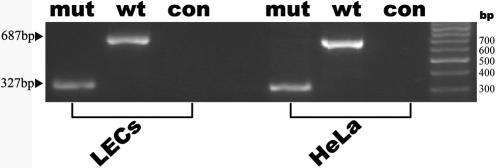
RT–PCR products obtained from minigene constructs transient transfection. The mutated (pTARGET-mut) and wild-type (pTARGET-wt) minigene constructs were independently transfected into HeLa cells and human lens epithelial cells (LECs). Forty-eight hours later, total RNA was extracted from transfected cells and analyzed by RT–PCR. The RT–PCR product obtained from pTARGET-wt and pTARGET-mut shows a 687 bp band and a 327 bp band, respectively. The pTARGET-col used as a control shows a negative band.

**Figure 3:**
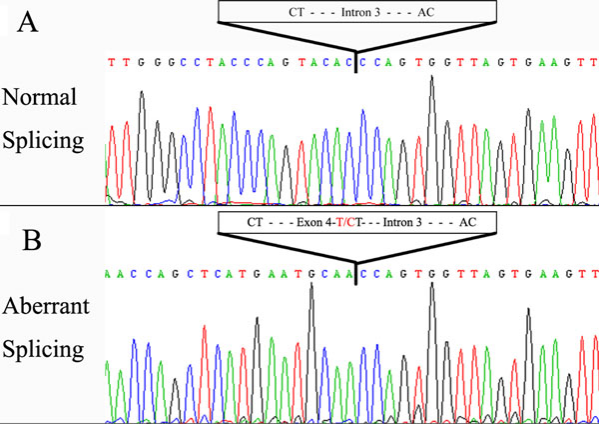
Reverse sequence of the wild-type and the mutant RT–PCR products. **A**: Normal splicing shows the exact excision of intron 3. **B**: Aberrant splicing shows the activation of a cryptic splice acceptor site within the 3′ UTR of the *MIP* gene, resulting in the loss of the coding region and most of the untranslated region of exon 4.

**Figure 4:**
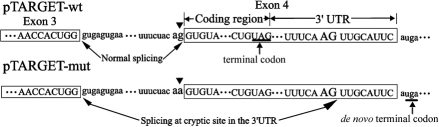
Schematic diagram of comparison between normal splicing and aberrant splicing. The upper panel shows the normal splicing of the wild-type RNA transcript. The lower panel shows that the G>A mutation (black triangles) at the acceptor splice site of intron 3 leads to activation of a cryptic acceptor splice site within the 3′ UTR of the *MIP* gene, resulting in the loss of the coding region and most of the untranslated region of exon 4. The underlining codon (UAG and uga) denote the wild type and de novo terminal codon, respectively. The 61 amino acids encoded by exon 4 are replaced by a Leu-His-Ser tripeptide.

Figure 2 showed the results when plasmids, pTARGET-mut, pTARGET-wt, and pTARGET-con were independently transfected to HeLa cells and LECs (not expressing AQP0), and total RNA was extracted from transfected cells and analyzed by RT–PCR followed by electrophoresis on 2% agarose gels. RT–PCR of the total RNA obtained from cells transfected by pTARGET-wt yielded a 687 bp band consistent with the correct splicing of pre-mRNA. This was confirmed by sequencing of the isolated PCR product (Figure 3A). In contrast, a 327 bp band was detected in cells transfected with pTARGET-mut (Figure 2) and the sequencing analysis shown in Figure 3B demonstrated that a cryptic acceptor splice site was activated at nt359 of exon 4 (c.965–966) in the 3′UTR of the *MIP* gene. A summary of these results was shown in Figure 4.

### Comparison of wild-type and mutant AQP0 structural models

**Figure 5:**
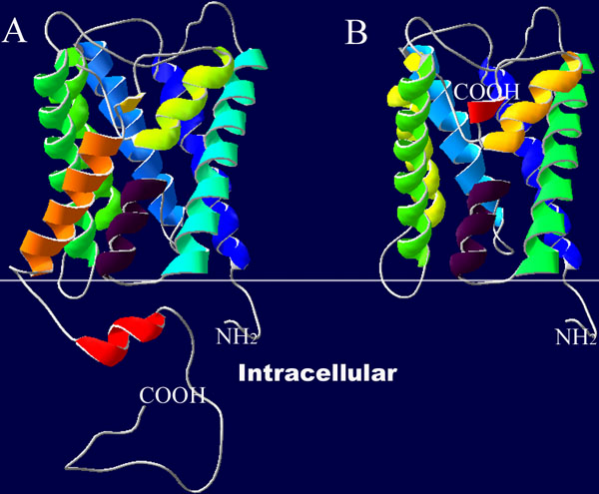
Comparison of wild-type and mutant AQP0 structural models by SWISS-MODEL. **A**: The wild-type AQP0 structural model contains six transmembrane α-helical domains with a short 9 amino acid NH_2_-terminus and a longer 39 amino acid COOH-terminus extending into the cytoplasm. **B**: The mutant AQP0 structural model shows the absence of the last α-helical domain as well as the complete COOH-terminus.

A mutant protein with 205 amino acids was supposed to be generated when the cryptic splice site in the 3′ UTR was activated. Wild-type AQP0 structural model contains six transmembrane α-helical domains with a short 9 amino acid NH_2_-terminus and a longer 39 amino acid COOH-terminus extending into the cytoplasm, whereas the mutant AQP0 structural model showed the loss of the last α-helical domain as well as the complete COOH-terminus. Instead, the aberrant AQP0 model (AQP0-LHS) was ended up having the Leu-His-Ser tripeptide (Figure 5).

## Discussion

It is reported that mutations in acceptor splice sites can result in exon skipping or activation of cryptic sites, either upstream or downstream of authentic sites [16]. In the present study, there was a mutation in the invariant AG dinucleotide of the last intron, so none of the existing canonical AG sites downstream are available at the 5′ border of following exon as an alternative acceptor splice site. According to previous studies, it is advisable to identify possible splicing defects by analyzing the mRNA available from the individual patients [17]. Therefore, we collected blood specimens and the cataracts from the proband during surgery. However, we failed to extract *MIP* mRNA from the blood specimens or the cataract probably due to the lower mRNA expression levels in the mutant alleles (unpublished). To confirm the role of this mutation, we finally made use of a simple minigene splicing assay [18]. Subsequently, we demonstrated that the acceptor splice-site mutation (c.607–1G>A) resulted in activation of a cryptic splice site in the 3′ UTR of the *MIP* gene. To our knowledge, cryptic acceptor splice site in the 3′ UTR has not been reported in human disease genes to date (summery in Table 1) [12,16]. According to Human Splicing Finder [19], the natural acceptor splice site is scored 96.46, while the cryptic acceptor splice site is scored 74.48. Interestingly, there is a consensus motif (TCTTTC) upstream of the acceptor splice site. Further study is required to determine whether there is a role for the TCTTTC motif in the aberrant splicing.

**Table 1:** Activation of aberrant 3′ splice sites in terminal introns/exons.

**Gene**	**Phenotype**	**Mutation**	**Location of cryptic 3′ ss**	**Location of stop codon**
*CLN3*	Batten disease	IVS15–1G>T	E15+7	E15+120
*G6PC*	Glycogen storage disease type 1a	E5+86G>T	E5+91	E5+512
*HPRT1*	Hypoxanthine-guanine phosphoribosyltransferase deficiency	IVS9–1G>A	E10+17	E10+48
*HPRT2*	Hypoxanthine-guanine phosphoribosyltransferase deficiency	IVS9–2A>G	E10+17	E10+48
*HPRT3*	Hypoxanthine-guanine phosphoribosyltransferase deficiency	IVS9–2A>T	E10+17	E10+48
*CD40LG*	X-linked hyper-IgM syndrome	IVS4–2A>G	E5+8	E5+377
*UGT1A1*	Crigler-Najjar syndrome type 1	IVS4–1G>A	E5+7	E5+298
*APOE*	ApoE deficiency	IVS3–2A>G	IVS3–52	E4+718
*ERCC3*	Xeroderma pigmentosum	IVS14–6C>A	IVS14–4	E15+132
*HBB*	Beta-thalassaemia	IVS2-A>G	IVS2–271	E3+129
*L1CAM*	X-inked hydrocephalus, MASA syndrome, spastic paraplegia	IVS26–12G>A	IVS26–10	E27+232
*MLYCD*	Malonyl-CoA decarboxylase deficiency	IVS4–14A>G	IVS4–13	E5+534
*MPO*	Myeloperoxidase deficiency	IVS11–2A>C	IVS11–109	E12+208
*SALL1*	Townes-Brocks syndrome	IVS2–19T>A	IVS2–17	E3+441
*SOD1*	Amyotrophic lateral sclerosis	IVS4–10T>G	IVS4–9	E5+108
*TPMT*	Thiopurine methyltransferase deficiency	IVS9–1G>A	IVS9+1, IVS9–330	E10+113

The activation of a cryptic acceptor splice site may lead to substitution of the distal end of the 6th trans-membrane domain and the COOH-terminal domain of AQP0 for a Leu-His-Ser tripeptide (AQP0-LHS). Previous studies have demonstrated that the AQP0 COOH-terminus is crucial for both lens development and transparency because of its interactions with calmodulin [20], the cytoskeletal proteins filensin and CP49 [21], and connexin 45.6 [22]. Cleavage of the intracellular COOH-terminus decreases water permeability [23] and enhances the adhesive properties of the extracellular surface of AQP0, indicating a conformational change in the molecule [24] such that AQP0-LHS may fail to form a functional channel. As a result, the internal homeostasis of the lens necessary to maintain transparency is disrupted, resulting in cataract formation.

In conclusion, analysis using an in vitro minigene system demonstrates that the *MIP* c.607–1G>A mutation leads to aberrant splicing by activation of a cryptic acceptor splice site in the 3′ UTR of the *MIP* gene. To our knowledge, this is the first report of cryptic acceptor splice site in the 3′ UTR of a human disease gene. The c.607–1G>A mutation creates a de novo AQP0-LHS, which is predicted to cause congenital cataracts by disrupting the internal homeostasis of the lens fiber cells.
